# Variations in the *OsGGP* uORF Fine-Tune Vitamin C Content and Confer Resistance to Osmotic Stress in Rice

**DOI:** 10.1186/s12284-025-00848-7

**Published:** 2025-10-27

**Authors:** Shifei Sang, Tengyun Ma, Yijing Zhang, Shiqian Zhang, Yanan Wang, Jinxia Zhang, Guoqin Yao, Liuchun Feng, Shengdong Ji, Hongtao Cheng, Junhua Li, Peng Yan

**Affiliations:** 1 College of Life Sciences, Henan Normal University/Engineering Research Center of Crop Genetic Improvement and Germplasm Innovation in Henan Province, No.46 Jianshe East Road, Muye, Xinxiang, 453007 Henan People’s Republic of China; 2 Institute of Crop Sciences, Chinese·Academy of Agricultural Sciences/Key Laboratory of Crop·Eco-physiology and Cultivation,·Ministry of·Agriculture and·Rural Affairs, Beijing, 100081 People’s Republic of China; 3 Oil Crops Research Institute of Chinese Academy of Agricultural Sciences/Key Laboratory for Biological Sciences and Genetic Improvement of Oil Crops, Ministry of Agriculture and Rural Affairs, Wuhan, 430062 People’s Republic of China; 4 Xinxiang Academy of Agricultural Sciences, Xinxiang, 453000 Henan People’s Republic of China

**Keywords:** Rice, Ascorbic acid, Upstream open reading frame, *GGP*

## Abstract

GDP-L-galactose phosphorylase (GGP) is a key rate-limiting enzyme in the ascorbic acid synthesis pathway, and it plays a crucial role in regulating ascorbic acid metabolism and redox balance. The upstream open reading frame (uORF) in the 5' untranslated region of the *GGP* gene typically suppresses translation efficiency, reducing vitamin C content. In this study, *GGP* uORF in rice was identified, and seven *GGP* uORF allele variants AL1-AL7 were generated by CRISPR/Cas9 technology; then, the effects of these variations on AsA content and osmotic stress resistance were evaluated. The AsA content of the seven mutant plants was 1.80-3.08 times greater than that of the control, and the increase of AL4 mutant was the greatest. Protoplast assays confirmed that the *OsGGP* uORF inhibits downstream ORF translation. Osmotic stress experiments revealed that in the mutant lines, both the activities of enzymes involved in reactive oxygen species (ROS) scavenging and the proline (Pro) content were significantly increased, while the levels of peroxides were significantly decreased. These results demonstrate that mutation of the *GGP* uORF significantly increases translation efficiency, AsA content, and the ability of rice to reduce ROS levels, restore osmotic balance, and improve ROS scavenging capacity under osmotic stress. This study reports the regulated osmotic stress of the *GGP* uORF in monocotyledonous plants for the first time and has created a variety of allelic variation germplasm resources. It provides a novel approach for improving AsA content and tolerance to salt stress in rice and other monocots via genetic approaches.

**Supplementary Information:**

The online version contains supplementary material available at 10.1186/s12284-025-00848-7.

## Introduction

Rice (*Oryza sativa* L.) is both a vital food crop and one of the most prominent model crops in agricultural research. Moreover, rice is the staple food for more than half of the population worldwide, and its by-products are crucial raw materials for animal feed and organic fertilizers, among others (Zeng et al. 2017). However, the exposure of rice to abiotic stress factors during cultivation can decrease crop yield. Thus, increasing the resistance of rice to such abiotic stress factors is crucial for maintaining high and stable yields, and this is a pivotal strategy for ensuring food security and promoting stable socio-economic development (Bai et al. 2018).

Vitamin C, which is also known as ascorbic acid (AsA), is a water-soluble vitamin that is primarily stored in its reduced form within chloroplasts under normal physiological conditions. AsA plays a crucial antioxidant role in plants (Jomova et al. 2024). Furthermore, a cofactor for enzymes, AsA participates in cell division and growth (Ye et al. 2012). Additionally, AsA functions as a vital antioxidant that enhances plant resistance to stress. In response to abiotic stress factors, plants produce various toxic substances, including singlet oxygen (^1^O_2_), superoxide radicals (O_2_^−^), hydrogen peroxide (H_2_O_2_), and hydroxyl radicals (·OH). These compounds can readily oxidize DNA, proteins, and lipids, damaging cellular functions (Broad et al. 2020). Antioxidant mechanisms in plants involve both enzymatic and nonenzymatic components, which help maintain the dynamic balance of ROS. These components mitigate excessive ROS production and oxidative stress, thereby preventing cellular damage. AsA donates electrons to a variety of enzymatic and nonenzymatic reactions, playing a significant role in antioxidant defense (Das et al. 2014, Alhagdow et al. 2017). For example, in the ascorbate‒glutathione cycle, reduced ascorbate reacts with H_2_O_2_, which is catalyzed by ascorbate peroxidase (APX), to produce monodehydroascorbate (MDHA); then, MDHA can be reduced back to ascorbate by monodehydroascorbate reductase (MDHAR). This process not only detoxifies H_2_O_2_ but also regulates ascorbate levels and other ascorbate biosynthetic pathways (Yin et al. 2010). Moreover, AsA directly scavenges ROS by neutralizing radicals through electron donation, effectively eliminating toxic radicals and other ROS that are generated during cellular metabolism and thus increasing resistance to abiotic stresses (Arrigoni et al. 2002). Consequently, increasing endogenous AsA levels in plants is a reliable approach for increasing the resistance of rice to abiotic stresses.

In vivo, ROS scavenging via AsA can be achieved by administering exogenous AsA or by increasing endogenous AsA levels, and both strategies increase the ability of plants to cope with various abiotic stresses (Gallie et al. 2013). For example, the overexpression of two *GME* gene family members in tomatoes significantly increases AsA accumulation, which is correlated with increased tolerance to stress under conditions of methyl viologen, cold, drought, and salt stress (Zhang et al. 2011a, b). Additionally, the combination of AsA and the endophytic nitrogen-fixing bacterium *Avi2* increases the photosynthetic efficiency, antioxidant capacity, and growth of rice under drought stress conditions (Kumar et al. 2021). Moreover, Ali et al. (2019) expressed *GGP* (encoding GDP-L-galactose phosphorylase) from kiwifruit in the widely cultivated indica rice variety IR64, leading to increased AsA levels and tolerance to multiple stress conditions without adverse effects on the agronomic traits of IR64. Conversely, suppressing *OsVTC1-3* (encoding GDP-mannose pyrophosphate synthetase) expression in rice plants results in rapid O^2−^ production and substantial H_2_O_2_ accumulation in the roots under salt stress conditions, significantly impairing plant tolerance to salt. However, exogenous AsA supplementation can restore salt tolerance in these plants, indicating that endogenous AsA is crucial for the response of rice to abiotic stress (Wang et al. 2018). Thus, increasing the AsA content in plants not only increases resistance to salt stress but also results in vitamin C-rich fruits, which have beneficial effects on human health.

**Fig. 1:**
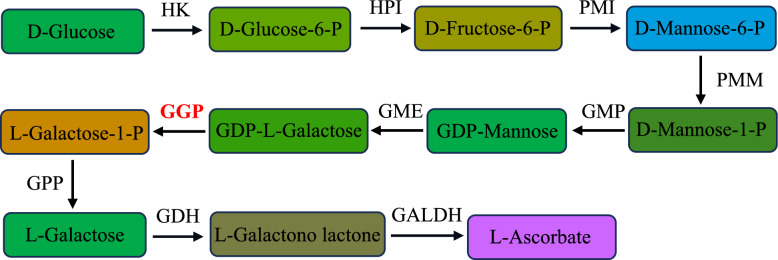
The L-Galactose pathway in ascorbate biosynthesis of plants. HK, hexokinase; HPI, hexose phosphate isomerase​; PMI, phosphomannose isomerase; PMM, phosphomanno mutase; GMP, GDP mannose pyrophosphorylase; GME, GDP mannose epimerase; GGP, GDP galactose phosphorylase; GPP, galactose-1-P phosphatase; GDH, galactose dehydrogenase; GALDH, galactono lactone dehydrogenase

AsA synthesis in plants is a complex physiological process that involves numerous biochemical reaction steps (Wheeler et al. 1998; Laing et al. 2015). GDP-L-galactose phosphorylase (GGP) catalyzes the conversion of GDP-L-galactose to GGP in the L-galactose pathway (Wheeler et al. 1998; Fig. 1). Numerous studies have demonstrated that GGP serves as a pivotal rate-limiting enzyme in the AsA biosynthetic pathway in plants, playing crucial roles in regulating AsA synthesis and metabolism and maintaining redox balance in the plant (Alhagdow et al. 2017). Wang et al. (2014) isolated the *GGP* gene from tomato leaves and successfully transferred it to tobacco, resulting in increased AsA contents and increased tolerance to cold stress in the transgenic tobacco plants. Conversely, suppressing *VTC2* (*GGP*) gene expression in plants dramatically reduces AsA levels and decreases stress resistance (Wang et al. 2013). Dowdle et al. (2007) reported that when the *GGP* gene was mutated along with either of the *VTC2* (*GGP*) and *VTC5* (encodes a second GGP with similar properties to VTC2) and homologous genes in *Arabidopsis thaliana*, the plants ceased to grow immediately after germination, leading to cotyledon bleaching. The normal growth of these mutant plants could be restored only through the exogenous application of AsA. Furthermore, when the *GGP* gene alone was mutated, the AsA content decreased by half. These findings highlight the significant role of the *GGP* gene in AsA synthesis and suggest that regulating *GGP* expression can effectively control AsA levels in plants.

Gene expression is regulated by a variety of *cis*-elements. By identifying the core *cis*-elements that regulate gene expression and making targeted modifications to these elements, desirable traits can be precisely controlled. For example, in tomatoes, extensive *cis*-regulatory mutations were generated by editing the *cis*-acting elements in the *CLAVATA3* gene promoter, and these mutations allowed the fine-tuning of tomato petal and locule numbers (Rodriguez et al. 2017). Moreover, the targeted editing and deletion of *cis*-acting elements in the rice *SBEIIb* promoter yielded germplasm resources with a range of resistant starch contents that met various application needs (Sang et al. 2025). Furthermore, regulation of mRNA translation, particularly during translation initiation, directly limits the rate of protein synthesis. Eukaryotic mRNAs consist of a 5' untranslated region (UTR), an open reading frame (ORF) that encodes the protein, and a 3' UTR (Wang et al. 2024). Among these regions, upstream open reading frames (uORFs) that are located within the 5' UTR are key *cis*-elements that affect translation efficiency, often inhibiting initiation of downstream primary open reading frame (pORF) translation by sequestering or competing for ribosomes (Zhang et al. 2019; Si et al. 2020). Notably, uORFs are prevalent in both the animal and plant kingdoms; 49% and 44% of total transcripts in humans and mice contain uORFs, respectively, and 20% to 40% of total transcripts in plants, such as rice, *A. thaliana*, and maize, contain uORFs (Um et al. 2021). Studies have demonstrated that the translation of an uORF can affect the gene expression of the subsequent main ORF, either by initiating mRNA decay or by modulating translation (Barbosa et al. 2013; Hinnebusch et al. 2016). For example, the conserved uORF in *FvebZIPs1.3* of strawberries encodes a peptide that influences the translation of downstream genes, subsequently affecting sugar metabolite production (Xing et al. 2020).

Laing et al. (2015) identified such a mechanism that regulates the synthesis of AsA in *A. thaliana*. When AsA concentrations are high, the uORF of the key AsA synthesis gene *GGP* inhibits the translation of *GGP*, whereas when AsA concentrations are low, *GGP* can be translated normally. These findings suggest that *A. thaliana* regulates *GGP* gene translation through a feedback regulatory mechanism to control AsA levels. Using this mechanism, several studies have reported the presence of a conserved uORF in the 5' UTR of *GGP* mRNA in dicotyledonous plants, such as *A. thaliana*, tomato, and lettuce, and this uORF inhibits the translation of the downstream pORF and thereby regulates AsA content (Laing et al. 2015; Zhang et al. 2018; Deslous et al. 2021; Li et al. 2018). To date, all reported *GGP* uORFs have been found in dicotyledonous plants. However, there is no evidence for the existence of a similar feedback mechanism that regulates AsA synthesis in monocotyledonous plants, such as rice, maize, and wheat.

In this study, we identified a conserved uORF of the key *GGP* gene that is involved in the L-galactose pathway of AsA synthesis in rice, and this uORF affects the translational efficiency of this gene. We subsequently evaluated the function of this uORF using a dual-luciferase reporter system. Additionally, the uORF was knocked out using CRISPR/Cas9 technology to generate germplasm resources with significantly increased resistance to salt stress. This study not only elucidates the function of the *GGP* uORF but also provides new genetic approaches for generating abiotic stress-resistant germplasms by utilizing allelic variations in the *GGP* uORF.

## Results

### Identification and Analysis of the GGP uORF Sequence in Rice

**Fig. 2:**
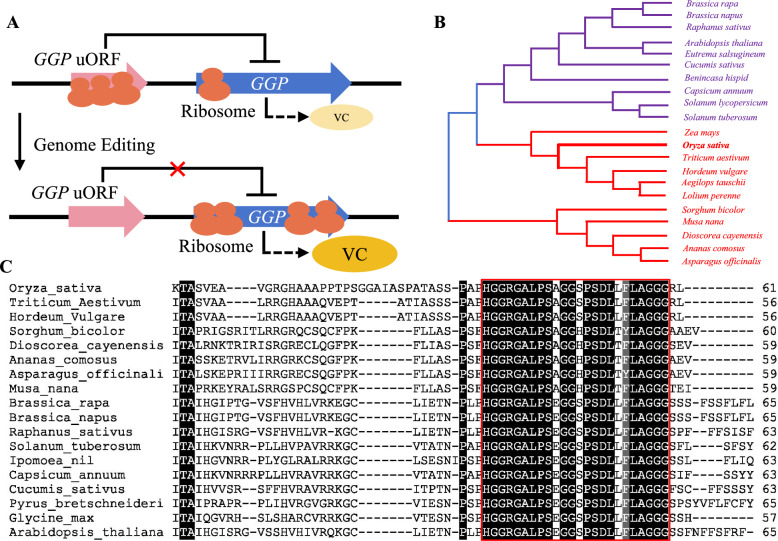
Mechanisms of action of *GGP* uORFs and identification of candidate *GGP* uORFs in Rice. **A** The currently reported functional schematic diagram depicts the uORFs of the *GGP* gene in dicotyledonous plants. It highlights the AsA-dependent post-transcriptional regulation of the *GGP* gene and introduces a strategy to enhance AsA content by engineering conserved AsA regulatory elements to control the uORF. By mutating the AsA-controlled uORF, the inhibition of *GGP* gene translation is reduced, resulting in increased accumulation of AsA. **B** Phylogenetic tree of *GGP* uORF in different species. **C** Alignment of the conserved *GGP* uORF amino acid sequences in dicotyledonous and monocotyledonous plants. Black box with white letter, 100% identity

Previous studies have shown that the sequence and function of the *GGP* uORF is conserved in dicotyledonous plants (Laing et al. 2015). The *GGP* uORF, which is located in the 5' UTR, can competitively bind to ribosomes, reducing the efficiency of ribosome binding to the pORF, which encodes *GGP*, thus inhibiting the translation of the pORF (*GGP*). However, knocking out the *GGP* uORF via gene editing techniques can alleviate the inhibition of *GGP* translation (Fig. 2A). By utilizing the reported amino acid sequence of the *AtGGP* uORF as a query sequence, potential rice *GGP* uORFs were retrieved from the GenBank database. The full-length sequence of the rice *GGP* uORF is 183 bp long, and in encodes 60 amino acids. Similar to the *GGP* uORFs that are found in dicotyledonous plants, the rice *GGP* uORF also features the unconventional start codon ACG. Then, *GGP* uORF sequences from various plants were identified via a comparable method, followed by phylogenetic tree construction and sequence alignment, and it was found that *GGP* uORFs from monocotyledonous and dicotyledonous plants share a common evolutionary origin (Fig. 2B). The *GGP* uORFs of monocotyledonous plants exhibit high sequence identity, indicating that sequence conservation occurs not only in dicots but also in monocots. Moreover, a conserved domain is present in both groups (Fig. 2C). These findings suggest that a conserved uORF is present in the *GGP* of monocotyledonous plants such as rice, and this conserved uORF can inhibit *GGP* expression.

### Mutation of the Rice GGP uORF Increases pORF Translation Efficiency

**Fig. 3:**
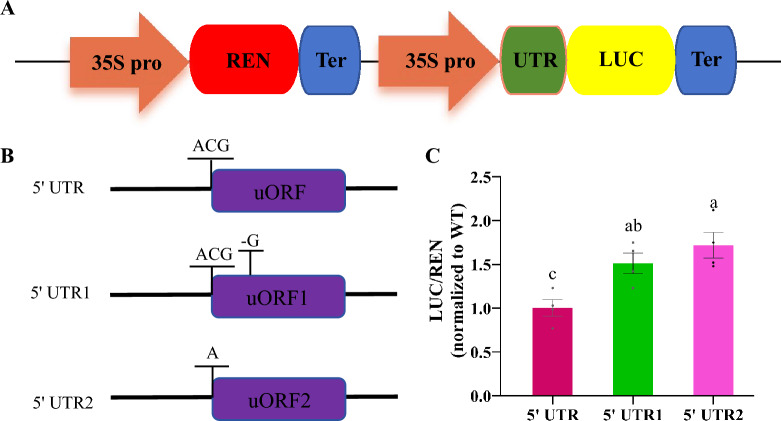
Effect of the artificially created *GGP* uORF allele variations on translation of the downstream primary open reading frame. **A** Schematic of the dual-luciferase reporter vector. 35S pro, cauliflower mosaic virus 35S promoter. REN, Renilla reniformis luciferase. LUC, luciferase. **B** The uORF allele variations was artificially created at the position of *GGP* 5' UTR. **C** Effect of the novel alleles on translation of the pORF in the dual-luciferase reporter system. In **C**, mean values (±SEM) are compared to those for wild-type using Student’s *t*-tests, *P* < 0.05

To investigate whether the *OsGGP* uORF can decrease the expression of the downstream pORF, we artificially mutated the uORF within the 5' UTR of the *GGP* gene and cloned the mutated UTR sequence into pGreenII0800 (Fig. 3A). The effects of the *GGP* uORF mutations on the translation efficiency of the downstream pORF were evaluated by determining the ratio of luciferase (LUC) expression to renilla luciferase (REN) expression. The results revealed that the LUC/REN ratios in protoplasts that were transformed with vectors carrying the mutant UTR1 and UTR2 sequences were 1.48 times and 1.83 times greater, respectively, than those of the WT protoplasts (Fig. 3B and C). Mutations both at the start codon position and in the central region of the *OsGGP* uORF significantly increased the LUC/REN ratio, indicating that *GGP* uORF mutations substantially increase the translation efficiency of the downstream pORF. It is hypothesized that the rice *GGP* uORF performs functions similar to those of *GGP* uORFs in dicotyledonous plants, such as *A. thaliana* and lettuce, and that mutating the rice *GGP* uORF may increase the AsA content in rice.

### Engineering of the Conserved uORF of GGP via CRISPR/Cas9

**Fig. 4:**
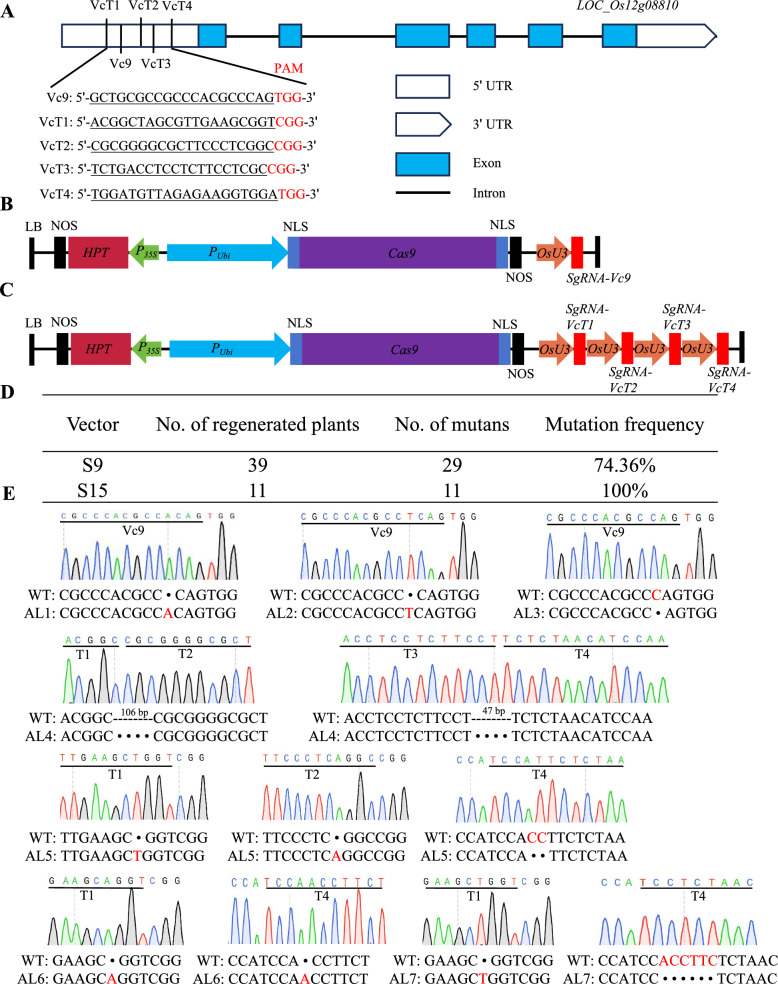
Engineering the uORF of *GGP* using CRISPR/Cas9. **A** Diagram of the uORFs in *GGP* and the target of CRISPR/Cas9. **B** and **C** Schematic of the edit vector. pUbi, maize *Ubiquitin-1* (*Ubi-1*) gene promoter. **D** Frequencies of mutations induced by CRISPR/Cas9 in T_0_ rice plants. **E** Sanger sequencing peak map of T_1_ generation *GGP* uORF edited homozygous single plant target sites

Given that mutations in the uORF of *OsGGP* increase the translational efficiency of the downstream pORF, we used CRISPR/Cas9 gene editing technology to modify the *OsGGP* uORF, aiming to develop germplasm resources enriched with AsA. Specifically, we selected five target sites—Vc9, VcT1, VcT2, VcT3, and VcT4—within the 5' UTR of *OsGGP* (Fig. 4A). We constructed both single-target editing vectors, such as S9 (which includes the Vc9 target site), and a multitarget editing vector, S15 (which includes VcT1, VcT2, VcT3, and VcT4) (Fig. 4B and C).

Genetic transformation was conducted with the conventional japonica rice variety Hongdao 59 as the recipient, and the *GGP* uORF editing status of the resulting regenerated seedlings was assessed. The results indicated that among the 39 positive T_0_ generation plants transformed with vector S9, 29 plants exhibited successful editing, yielding an editing rate of approximately 74.36% (Fig. 4D). In contrast, all 11 T_0_ generation edited plants transformed with vector S15 exhibited successful mutations, resulting in a nearly 100% editing rate. Notably, mutations in S9-transformed regenerated individuals were primarily single-base insertions or deletions. In comparison, the S15-transformed regenerated plants presented a broader spectrum of editing types, including large fragment deletions, base insertions, deletions, and other mutation types (Fig. 4E).

On the basis of the identified mutations, we classified the types of *GGP* uORF mutations into seven allelic variations, namely, AL1 through AL7. Allelic variations AL1 to AL3, which were found in the S9-transformed seedlings, featured frameshifts attributed to base insertions or deletions (Fig. 4E). Variations AL4 to AL7, which were observed in plants transformed with vector S15, included a 106-bp deletion between T1 and T2 and a 47-bp deletion between T3 and T4 for AL4. The AL5 variant displayed base insertions or deletions at T1, T2, and T4, whereas both the AL6 and AL7 mutations affected only the T1 and T4 target sites (Fig. 4E). Collectively, these results indicate the successful acquisition of a diverse array of allelic variations for the *GGP* gene.

### Impact of New Alleles on the Translational Efficiency of the pORF

**Fig. 5:**
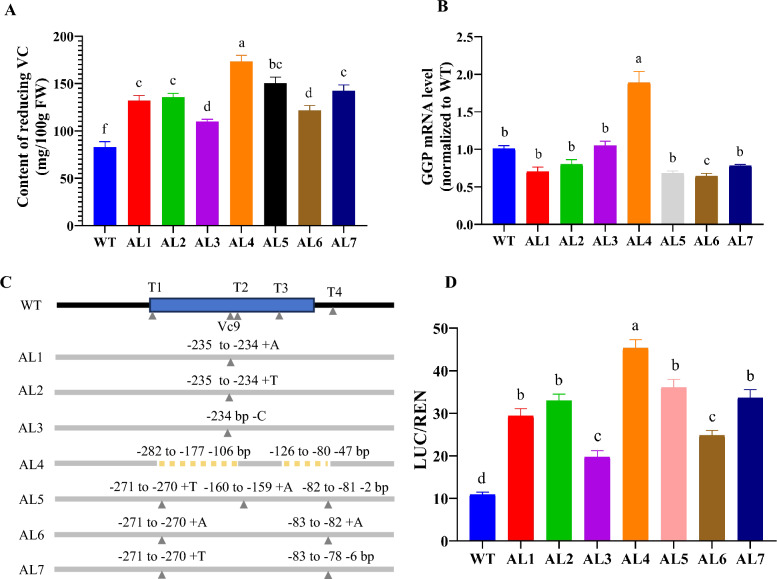
The impact of novel alleles on vitamin C content and downstream primary open reading framework translation in rice. **A** The effect of new allele variation on the AsA content in rice. **B** The effect of new allelic variations on the expression level of *GGP* gene in rice. **C** Schematic diagram of different allelic variations at the *GGP* 5'UTR position. **D** Insert *GGP* 5'UTR with different allelic variations into a dual luciferase expression vector and detect the LUC/REN ratio. In **B** and **C**, mean values (±SEM) are compared to those for wild-type using Student’s *t*-tests, *P* < 0.05

To further investigate the effects of mutations in the rice *GGP* uORF on AsA levels, we measured the AsA content in plants with various *GGP* uORF allelic variations. Compared with WT plants, all the allelic variations exhibited significantly increased AsA levels. Specifically, compared with those in the control plants, the AsA levels in the leaves of AL1 through AL7 were increased by 58.77%, 62.97%, 32.17%, 108.65%, 80.60%, 46.59%, and 71.56%, respectively (Fig. 5A). Notably, the AL4 variant, which exhibited a large fragment deletion in the *GGP* 5' UTR, presented the greatest increase in AsA content, suggesting that deleting the *GGP* uORF and shortening the *GGP* 5' UTR length is an effective approach for improving the AsA content.

We subsequently examined the mRNA expression levels of the *GGP* gene across various allelic variations. The results revealed significant differences in *GGP* expression only in the leaves of AL4 and AL6 compared to WT. In contrast, no significant differences were observed in the *GGP *expression levels in the leaves of AL1, AL2, AL3, AL5, and AL7 compared to WT (Fig. 5B). This downregulation may occur due to the increased AsA levels in the mutants, which inhibited GGP gene mRNA transcription to a certain extent. The increased GGP expression in AL4 leaves could be related to the large fragment deletion in the UTR, which among all the allelic variations, resulted in the highest AsA contents.

Despite the lack of significant increases in *GGP* mRNA expression among the various allelic variations, the AsA contents increased considerably. This finding led us to hypothesize that the increase in AsA levels might occur due to increased *GGP* translation efficiency. To test this hypothesis, we amplified the *GGP* 5' UTR sequences of the AL1–AL7 and WT alleles by PCR and cloned these sequences into the dual-luciferase vector pGreenII0800 to evaluate the effect of each allelic variation on translation efficiency (Figs. 3A and 5C). The results showed that the protoplasts that were transformed with alleles AL1 through AL7 exhibited LUC/REN ratios that were increased by 1.69, 2.01, 0.80, 3.14, 2.29, 1.27, and 2.07 times, respectively, compared with that of the WT protoplasts. These findings indicate that mutations in the *OsGGP* uORF can increase the AsA contents in rice plants by improving *GGP* translation efficiency. In particular, the AL4 allele not only increased *GGP* gene expression but also enhanced its translation efficiency.

### Mutations in the GGP uORF Increase Salt Osmotic Stress

**Fig. 6:**
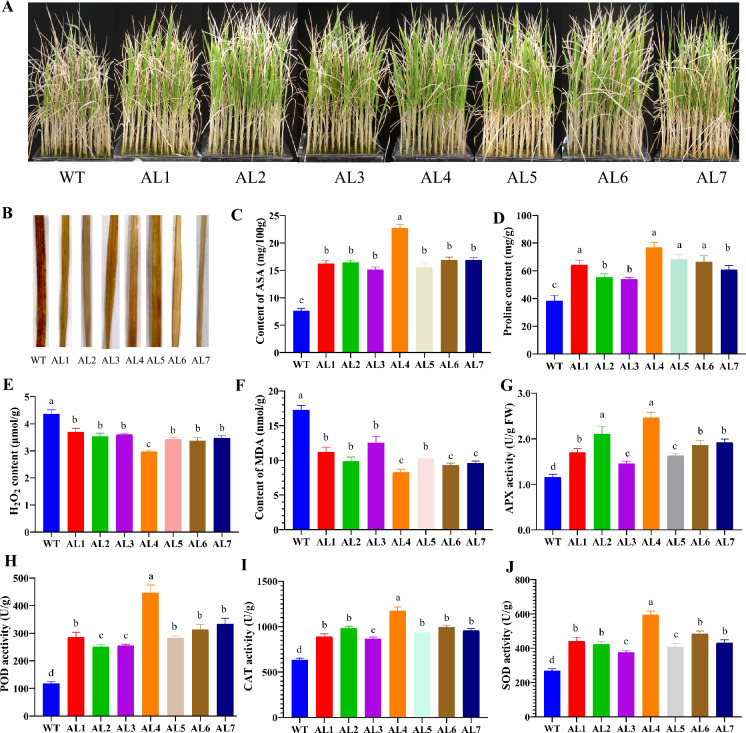
*GGP* uORF mutation in rice can enhance osmotic stress tolerance. **A** Identification of salt tolerance of different homozygous allelic mutants of *GGP* uORF by hydroponics. **B** Under osmotic stress, DAB identified the ROS content of different homozygous allelic mutant plants of *GGP* uORF. **C**–**F** The content of AsA, Pro, H_2_O_2_ and MDA in leaves of different homozygous mutant plants of *GGP* uORF under salt stress. **G**–**J** The activities of peroxidase APX, POD, CAT and SOD in leaves of different homozygous mutant *GGP* uORF plants under salt stress

AsA is a crucial antioxidant that significantly impacts the responses of plants to oxidative stress (Zhang et al. 2012). To explore how differences in AsA contents among different rice seedlings affect the tolerance of these seedlings to stress, we treated transformed seedlings, as well those with homozygous allelic variations (AL1-AL7), with 100 mmol/L NaCl to induce osmotic stress. After two weeks, many WT seedlings exhibited severe wilting, and some even died. In contrast, while some AL1-AL7 variant seedlings exhibited signs of stress, their wilting was notably less severe than that observed in the WT seedlings, indicating superior vigor under stress conditions. Specifically, the AL4 variant seedlings, which had increased AsA levels, demonstrated significantly better growth than the control group (Figs. 6A and S1). The 3,3'-Diaminobenzidine (DAB) method was used to evaluate the content of ROS in leaves of different allelic variation materials under osmotic stress. The results showed that compared with WT, the staining degree of *GGP* uORF editing strain was significantly weaker than that of the control, indicating that the editing strain cells had stronger ability to clear ROS (Fig. 6B).

Further measurements of physiological stress indicators revealed that the Pro and AsA contents in the stressed AL1-AL7 seedlings were significantly greater than those in the WT seedlings (Fig. 6B and C). Additionally, the AsA contents were 2.69, 3.01, 1.80, 4.14, 3.29, 2.27, and 3.07 times greater in the AL1-AL7 seedlings than those in the control seedlings, respectively (Fig. 6C). Additionally, the H_2_O_2_ and malondialdehyde (MDA) contents in the leaves of the AL1-AL7 stressed seedlings were significantly lower than those in the control seedlings (Fig. 6E and F). These findings suggest that the edited seedlings exhibited reduced oxidative stress and, therefore, were more tolerant to osmotic stress. Furthermore, the activities of various peroxidases, such as APX, peroxidase (POD), catalase (CAT), and superoxide dismutase (SOD), were significantly elevated in the leaves of AL1-AL7 stressed seedlings compared with those of WT seedlings (Fig. 6G–J). These results collectively demonstrate that increased levels of endogenous AsA in rice increase the activities of peroxidases, facilitating the scavenging of peroxides that are generated under stress conditions and improving the survival of plants.

### GGP uORF Mutations Do Not Affect Agronomic Traits

To investigate the effects of the AL1–AL7 allelic variations on agronomic traits, we conducted statistical analyses of several parameters, including plant height, flag leaf length, flag leaf width, panicle length, grain length, grain width, seed-setting rate, and 1000-grain weight. The results revealed that, with the exception of a notable decrease in 1000-grain weight of AL4 compared with WT, there were no significant differences in the other agronomic traits between AL1–AL7 rice and WT rice (Tables S1 and S2, Fig. S2). These findings clearly demonstrate that mutations in the *GGP* uORF do not alter agronomic performance, suggesting that the uORF may be an ideal target site for improving both AsA content and osmotic stress tolerance in rice.

## Discussion

Rice is a critical staple food crop worldwide; however, its yields are decreased when exposed to abiotic stresses. Consequently, improving the resistance of rice to these stresses is a key strategy for mitigating these adverse effects. AsA is known to be a significant antioxidant in plant cells, and increased endogenous AsA levels effectively augment the resistance of rice to abiotic stresses.

Research indicates that *GGP* uORF is a promising target site for regulating endogenous ascorbic acid levels. The initiation of the *GGP* uORF must occur at the noncanonical start codon ACG, which features a Kozak sequence and is conserved across all plants from mosses to angiosperms (Laing et al. 2015). Rice *GGP* uORF can lead to ribosome retention, thereby reducing the efficiency of GGP translation (Carey-Fung et al. 2024). Using homology alignment methods, we identified potential *GGP* uORF sequences in rice. Typically, mutations in an uORF sequence can significantly increase the expression of the downstream pORF, potentially leading to phenotypic variation among organisms (Occhi et al. 2013). In our study, we artificially mutated the uORF, integrated these mutations into a dual-luciferase vector, and confirmed that *GGP* uORF mutations can increase the translation efficiency of downstream pORFs. These findings suggest that site-directed mutagenesis of uORFs via gene editing can effectively alleviate downstream ORF inhibition, suggesting a viable method for increasing AsA content. Additionally, our results demonstrated that mutations in the *GGP* uORF significantly increased AsA levels in rice. We also found that large fragment deletions in the uORF region of the 5' UTR resulted in even higher AsA contents, potentially reflecting changes in gene expression levels.

The study revealed that following large fragment deletions, *GGP* gene expression was significantly upregulated compared with that of the WT, whereas single-base deletions and insertions in the uORF of the *GGP* gene, as well as small fragment deletions, did not increase *GGP* expression. However, regardless of which of the above-mentioned mutation types, all of them can significantly enhance the translation efficiency of the downstream open reading frame (pORF) and lead to a marked increase in ascorbic acid content. Previous studies on lettuce and tomato have suggested that large fragment deletions in an uORF lead to *GGP* gene upregulation, and this is hypothesized to occur because of the promotion of mRNA accumulation by large fragment deletions, increased *GGP* gene translation, or increased mRNA stability in the absence of uORF regulation (Zhang et al. 2018; Deslous et al. 2021). This study investigated the effects of different allelic variations in *GGP* uORF on the translation efficiency of downstream *GGP* genes, and elucidated that allelic variations increase rice AsA content by regulating *GGP* translation efficiency.

Rice is among the crops that are most susceptible to salt stress, and high Na^+^ accumulation has been proven to be negatively correlated with rice growth (Platten et al. 2013). Numerous studies have shown that salt stress significantly inhibits rice growth, leading to issues such as leaf senescence, reduced height, and impaired root development, which are often accompanied by increased ROS production (Chang et al. 2019). Under normal growth conditions, the intrinsic ROS-scavenging system of plants helps maintain ROS levels. However, salt stress conditions lead to excessive accumulation of ROS, including H_2_O_2_, O_2_^−^, and ·OH, which can damage cellular structures and impair plant health (Akram et al. 2017). AsA, which is a vital antioxidant that is present in plant tissues, plays a critical role in neutralizing ROS, thereby helping to regulate intracellular ROS levels and support the function and growth of normal cells (Mansoor et al. 2022). Previous studies have established the role of AsA in alleviating oxidative stress in various plant species, including *A. thaliana*, tomato, tobacco, and potato. For example, Shalata et al. (2001) demonstrated that under salt stress conditions, tomato seedlings exhibited increased lipid peroxidation in roots, stems, and leaves, but this effect was partially mitigated by the administration of exogenous AsA, which increased tolerance to salt and reduced lipid peroxidation. Li et al. (2016) reported that site-specific mutants in *VTC1* led to increased AsA accumulation and reduced H_2_O_2_ levels in transgenic *A. thaliana*, thus promoting seedling growth. Yin et al. (2010) reported that overexpressing dehydroascorbate reductase conferred tolerance to aluminum stress in transgenic tobacco. Additionally, Upadhyaya et al. (2009) reported that the overexpression of the strawberry *GalUR* gene resulted in a 1.6–2-fold increase in the AsA contents of transgenic potatoes, leading to improved survival rates under various abiotic stresses.

In this study, we evaluated the resistance of individual edited plants (AL1 to AL7) to salt osmotic stress. Our findings indicated that variant materials with increased endogenous AsA contents exhibited greater growth under osmotic stress conditions. These findings suggest a positive correlation between increased AsA levels and increased resistance to osmotic stress among the edited seedlings. Further analysis of physiological indicators under osmotic stress conditions revealed that the edited plants exhibited increased ROS-scavenging capacity, increased soluble osmolyte accumulation, activity of peroxidase, and reduced cellular damage. These findings demonstrate that the superior growth performance of the edited seedlings under stress conditions is attributed to their elevated AsA levels, which regulate the osmotic balance and ROS homeostasis, thereby mitigating salt-induced damage in rice seedlings.

Although multiple studies have reported the function of *GGP* uORF in dicotyledonous plants, research on *GGP* uORF in monocotyledonous plants remains relatively limited. To our knowledge, this study represents the first report of *GGP* uORF regulating osmotic stress tolerance in monocotyledonous plants. Furthermore, this study confirms that both the sequence and function of the *GGP* uORF are conserved not only in dicots but also in monocots. This research successfully generated multiple allelic variations of the *GGP* uORF and provided diverse germplasm materials that are rich in AsA and are capable of withstanding osmotic stress, thereby providing new genetic resources for the future enhancement of crop varieties.

## Materials and Methods

### Identification and Functional Analysis of uORFs in the Rice GGP Gene

We searched tblastn (BLAST: Basic Local Alignment Search Tool) (Altschul et al. 1990), using the reported amino acid sequence of the *A. thaliana GGP* uORF as the query sequence and limiting our search to rice species, to identify potential candidate ORFs. A similar approach was used to identify *GGP* uORF sequences from other monocotyledonous and dicotyledonous species. MEGA 7.0 was subsequently utilized to perform sequence alignment and evolutionary analysis of the identified *GGP* uORF sequences (Kumar et al. 2016). Then, the candidate rice *GGP* uORFs were subjected to site-directed mutagenesis. Next, both the wild-type and mutant *GGP* 5' UTR sequences were cloned and inserted into the dual-luciferase reporter vector pGreenII0800. With this vector, firefly luciferase gene expression was driven by the cauliflower mosaic virus 35S promoter (CAMV 35S promoter). These constructs were then transformed into rice protoplasts, and the LUC/REN ratio was determined using a fluorescence microplate reader (SH1M2F, Agilent Technologies Co., Ltd., U.S.A.) to evaluate the effects of the *GGP* uORF mutations on the translation efficiency of the downstream pORF (Zhang et al. 2011a, b). The primers used in the study are stored in Table S3.

### Construction and Genetic Transformation of Editing Expression Vectors Targeting OsGGP uORFs

On the basis of the DNA sequence of the *GGP* uORF, five editing target sites were designed to construct single-target and four-target editing vectors. The vector construction method followed that established by Ma et al. (2015) for CRISPR/Cas9-mediated editing of multiple plant genes. The resulting recombinant expression vectors were subsequently transformed into *Agrobacterium tumefaciens* EHA105. Genetic transformation of rice was conducted with the japonica rice variety Hongdao 59 as the transformation recipient. The target sequence for editing was amplified using PCR, and the PCR-amplified products were subjected to Sanger sequencing (Shanghai Shenggong Biotechnology Co., Ltd., Shanghai, China). The transformed and edited regenerated plants were cultivated at the transgenic experimental base of the College of Life Sciences, Henan Normal University. PCR amplification was conducted using DNA isolated from T1 plants, with the resultant amplicons submitted to Sangon Biotech (Shanghai) Co., Ltd. for Sanger sequencing to determine the mutation status of the T1 generation. Transgene-free plants were screened via PCR with primer pairs pYLF/pYLR and HygF/HygR; a plant was classified as non-transgenic only if both primer pairs generated negative PCR results.

### Extraction of Total mRNA and Quantification of Gene Expression Levels

mRNA was extracted from the leaves of Hongdao 59 and various homozygous allelic mutant lines using the TRIzol method (Jordon et al. 2015). Using this mRNA as a template, cDNA was synthesized with reverse transcriptase and random primers. qRT‒PCR was performed with the Hieff UNICON® Universal Blue qPCR SYBR Green Master Mix (Yisheng Biotechnology Co., Ltd., lot number HB220119) to determine gene expression levels. All gene expression analyses were performed with ​3 biological replicates​, with 3 technical replicates per biological sample to ensure reproducibility.

### Determination of AsA Content and Osmotic Stress Tolerance

The AsA content in the rice plants was determined by sampling the flag leaves according to the method described by Zhang et al. (2015). The AsA content of each mutant line was measured annually over two consecutive years, with three replicates for each mutant genotype in each year. After seed germination, rice seedlings were hydroponically cultured in Kimura B nutrient solution under the following conditions: temperature of 28±2 ℃, and a light regime of 12-h light/12-h dark photoperiod. (Zhang et al. 2024). At the three-week stage of seedling growth, salt stress induction treatment was conducted using 100 mM NaCl solution for two weeks. Following this treatment, samples were collected, and relevant physiological indicators were measured, each with 3 biological replicates. The contents of H_2_O_2_, PRO, and MDA, as well as the activities of APX, POD, CAT, and SOD, were analyzed using commercially available kits from Grace Biotechnology Co., Ltd. (Suzhou, China).

The H_2_O_2_ content was determined according to the ability of H_2_O_2_ to form a yellow complex with titanium sulfate, which exhibits an absorption peak at 415 nm. The Pro content was analyzed based on the ability of Pro to react with acidic ninhydrin to form a red product that absorbs at 520 nm, and its content was determined using a standard curve of known concentrations of pure Pro. The MDA content was measured on the basis of its reactivity with thiobarbituric acid (TBA); samples were treated with TBA to form a brownish-red trimethyldiketone product with a maximum absorption wavelength of 532 nm. The MDA content was calculated using differences in absorbance at 532 nm, 450 nm, and 600 nm. APX catalyzes the reaction between AsA and H_2_O_2_, oxidizing AsA. The absorbance at 290 nm decreases as AsA is oxidized, and APX activity was calculated on the basis of the decrease in absorbance per unit time. In the presence of H_2_O_2_, POD catalyzes the oxidation of guaiacol, producing a brownish-red tetramethoxyphenol product with a characteristic absorption peak at 470 nm, and its color intensity is proportional to its concentration within a certain range. Therefore, POD activity was estimated by spectrophotometrically measuring the absorbance of the reaction mixture. Catalase decomposes H_2_O_2_, leading to a decrease in the absorbance of a reaction solution at 240 nm over time, and CAT activity was calculated on the basisof the rate of change in absorbance. The activity of SOD was measured using the NBT method; NBT reacts with superoxide anions (O^2−^), which is catalyzed by xanthine oxidase (XO), to produce a colored substance that absorbs at 560 nm. SOD scavenges O^2−^, inhibiting the formation of this colored substance; thus, the darker the color of the reaction solution is, the lower the SOD activity is, and vice versa. 3,3'-Diaminobenzidine (DAB) is used for the qualitative analysis of reactive oxygen species (ROS) in plant leaves. After staining with DAB in the darkroom for 8 h, the leaves were decolorized with absolute ethanol until the green color was completely removed.

### Investigation and Statistical Analysis of Traits

When the plants reached maturity, we conducted statistical analyses on plant height, tiller number, panicle length, grain number per panicle, and grain length, with 3 biological replicates per trait and continuous tracking and measurement over two years; these analyses were conducted on wild-type (WT) plants as well as each edited rice plant mutant. Graph analyses and plotting were conducted using GraphPad Prism version 8.0. Student's *t*-test was used to identify significant differences among these traits. Analysis of variance multiple comparisons, different letters represent *p* < 0.05 level differences.

## Supplementary Information

Additional file 1

## Data Availability

The data that support the findings of this study are available within the Figure and Supplementary Data and Tables, or are available from the corresponding author upon request.
