# Supplementary material for: Variations in the OsGGP uORF Fine-Tune Vitamin C Content and Confer Resistance to Osmotic Stress in Rice
Source: Rice (N Y). 2025 Oct 27;18:98. doi: 10.1186/s12284-025-00848-7 (PMC12559526; doi:10.1186/s12284-025-00848-7)
Supplement: Supplementary file 1 — Additional file 1 [file 12284_2025_848_MOESM1_ESM.docx]

**Table S1 Analysis of plant type related traits of T_1_ generation edit plants**

| Types | Plant height (cm) | Flag leaf length (cm) | Flag leaf width (cm) | Panicle length (cm) | Tiller number |
| --- | --- | --- | --- | --- | --- |
| HD59 | 90.83±2.75 a | 28.03±1.93 a | 1.83±0.15 a | 16.70±0.80 a | 4±1 a |
| AL1 | 93.93±1.53 a | 27.80±2.04 a | 1.90±0.17 a | 16.80±0.92 a | 3±1 a |
| AL2 | 91.90±2.13 a | 28.53±2.32 a | 1.90±0.10 a | 17.13±0.68 a | 3±1 a |
| AL3 | 93.23±1.86 a | 29.03±1.70 a | 1.87±0.06 a | 17.47±0.61 a | 2±1 a |
| AL4 | 88.67±2.08 b | 27.73±1.21 a | 1.87±0.10 a | 17.20±0.78 a | 4±1 a |
| AL5 | 91.33±1.61 a | 26.77±2.70 a | 1.80±0.10 a | 17.93±0.93 a | 3±1 a |
| AL6 | 90.87±3.44 a | 26.73±1.55 a | 1.73±0.06 a | 17.10±0.96 a | 3±1 a |
| AL7 | 93.10±2.67 a | 26.40±2.10 a | 1.87±0.12 a | 17.40±1.28 a | 3±2 a |

**Table S2 Analysis of grain related traits of T_1_ generation editing plants**

| Types | Grain length (mm) | Grain width (mm) | Seed setting rate (%) | 1000 grain weight (g) |
| --- | --- | --- | --- | --- |
| HD59 | 7.59±0.26 a | 3.25±0.03 a | 74.67±2.52 a | 26.36±0.39 a |
| AL1 | 7.65±0.08 a | 3.29±0.09 a | 74.30±2.86 a | 26.73±0.49 a |
| AL2 | 7.67±0.25 a | 3.29±0.03 a | 79.27±4.30 a | 26.39±0.98 a |
| AL3 | 7.68±0.09 a | 3.22±0.06 a | 74.00±2.65 a | 26.77±1.01 a |
| AL4 | 7.89±0.14 a | 3.45±0.15 a | 72.87±2.80 a | 25.33±0.57 b |
| AL5 | 7.59±0.15 a | 3.35±0.08 a | 76.23±2.00 a | 26.37±0.45 a |
| AL6 | 7.85±0.17 a | 3.28±0.06 a | 74.87±2.42 a | 26.99±0.24 a |
| AL7 | 7.75±0.10 a | 3.36±0.08 a | 75.17±3.30 a | 27.26±0.44 a |

| **Table S3 The primer sequences used in this study** | | |
| --- | --- | --- |
| Primer Name | Sequence (5'-3') | Purpose |
| Vc9F | ggcaGCTGCGCCGCCCACGCCCAG | Single target vc9 edit vector target primer |
| Vc9R | aaacCTGGGCGTGGGCGGCGCAGC |  |
| VcT1F | ggcACGGCTAGCGTTGAAGCGGT | Multi-target editing primers |
| VcT1R | aaacACCGCTTCAACGCTAGCCG |  |
| VcT2F | ggcaCGCGGGGCGCTTCCCTCGGC |  |
| VcT2R | aaacGCCGAGGGAAGCGCCCCGCG |  |
| VcT3F | ggcaTCTGACCTCCTCTTCCTCGC |  |
| VcT3R | aaacGCGAGGAAGAGGAGGTCAGA |  |
| VcT4F | ggcaTGGATGTTAGAGAAGGTGGA |  |
| VcT4R | aaacTCCACCTTCTCTAACATCCA |  |
| HygF | GACGTAAGGGATGACGCACAATC | Positive identification of regenerated plants |
| HygR | GAACAGCGGGCAGTTCGGTTTCA |  |
| pYLF | TTATCGAGACGAACGGTGAG |  |
| pYLR | AAAGGTCGATACGAGTCTCG |  |
| ActinF | CCAAGGCCAATCGTGAGAAGA | qRT-PCR identification of *GGP* gene expression level |
| ActinR | AATCAGTGAGATCACGCCCAG |  |
| qGGPF | TGCCTCAGAGGATTGACCAGG |  |
| qGGPR | GTGGTTGATGGTTGCAAAGGC |  |
| VcideF | CTCGCCTACCACCACCACCA | Amplified target site sequence |
| VcideR | TGGATGTTAGAGAAGGTGGA |  |
| 35SlucF | gggcgaattgggtaccTGAGACTTTTCAACAAAGGGTAATT | Amplification of 35S promoter sequence and ligation of pgreen0800 vector |
| 35SlucR | tcccccactgggcgtTGTCCTCTCCAAATGAAATGAACTTC |  |
| uORFLUCF | catttggagaggacaagcttCTCGCCTACCACCACCACCA | Amplification of 5'UTR of wild-type GGP gene |
| uORFLUCR | gctctagaactagtggatccGATTAAATCTCAATTCCTTC |  |
| uORFLUCF | catttggagaggacaagcttCTCGCCTACCACCACCACCA | Amplified mutant GGP 5'UTR2 |
| uorf2R | CGACCGCTTCAACGCTAGCTCTTTGCCGCCTTTCAACTC |  |
| uorf2F | GAGTTGAAAGGCGGCAAAGAGCTAGCGTTGAAGCGGTCG |  |
| uORFLUCR | gctctagaactagtggatccGATTAAATCTCAATTCCTTC |  |
| uORFLUCF | catttggagaggacaagcttCTCGCCTACCACCACCACCA | Amplified mutant GGP 5'UTR1 |
| uorf1R | GATGGCTCCCCACTGGGCGTGGGCGGCGCAGCTGCGTGG |  |
| uorf1F | CCACGCAGCTGCGCCGCCCACGCCCAGTGGGGAGCCATC |  |
| uORFLUCR | gctctagaactagtggatccGATTAAATCTCAATTCCTTC |  |
| uORFLUCF | catttggagaggacaagcttCTCGCCTACCACCACCACCA | Amplified mutant plants *GGP* 5'UTR sequence |
| uORFLUCR | gctctagaactagtggatccGATTAAATCTCAATTCCTTC |  |


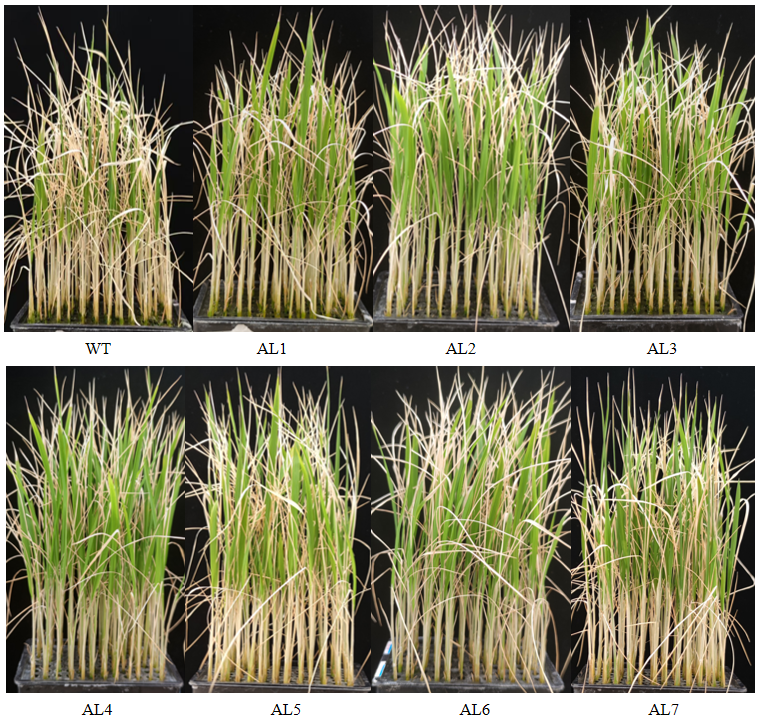


**Figure S1** Identification of osmotic tolerance of different homozygous allelic mutants of *GGP* uORF by hydroponics.


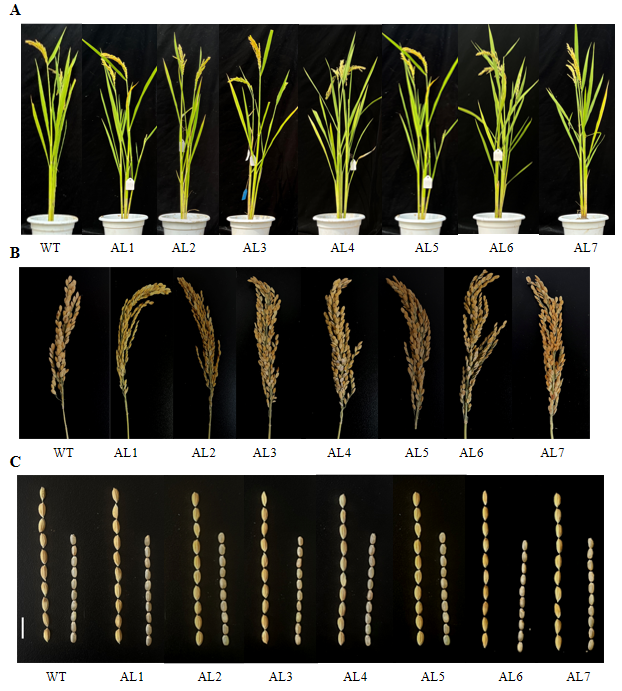


**Figure S2** Agronomic traits of different GGP uORF allelic variation plants. (A) Comparison of whole plants with different GGP uORF alleles. (B) Comparison of spikes of different GGP uORF allelic variation plants. (C) Comparison of grain traits of different GGP uORF allelic variation plants.
